# The San Antonio Board of Health Denies the Charges of Concealing the Presence of Meningitis in That City

**Published:** 1912-07

**Authors:** 


### Communications

### The San Antonio Board of Health Denies the Charges of Concealing the Presence of Meningitis in That City.

SAN ANTONIO, TEXAS, June 10, 1912.

*Editor Texas Medical Journal ("Red Back") Austin, Texas.*

DEAR SIR: In your June number of 1912, under the heading "A Serious Charge," you quote the *New York Medical Record* for May 4, 1912. [See June number.—EDITOR.]

You also, to our satisfaction, kindly add, that this charge is impossible, knowing, as you do, the gentlemen composing the San Antonio Board of Health. We assure you that you are not mistaken in your assumption. If that "wife of an army officer" would only have asked the medical director of the department of Texas, at the army post, Fort Sam Houston, she would not have published such an incorrect statement.

It is not true that the Board of Health did not allow cases of epidemic cerebro-spinal meningitis to be reported. The reporting of this disease is demanded by ordinance, and the profession at large has been admonished by special circulars and in the Bexar County Medical Association to report such cases promptly, and all possible means were taken in quarantining places where cases happened. Guards were stationed at such houses and placards were affixed in conspicuous places, and the houses, after the cases were over, were thoroughly fumigated. It is not true that most of the cases have originated in the schools, as only fourteen cases out of a total number of seventy-five cases up to date have happened among children frequenting public and private schools—five in private and nine in public schools. For this reason, and also after consulting with the State Health Officer, who advised against closing, schools were not closed. Schoolhouses were fumigated weekly. The medical director of the army post was promptly informed about every case that happened and was frequently present at the meetings of the Board of Health, as was the president of the school board. It is also incorrect to state that the army post was placed under effective quarantine, which would make one believe that no communication whatever was possible between the army post and the outside world. Only the coming of the soldiers in numbers and mingling with the people was restricted, and the coming to town of the soldiers was allowed by passes, and there was absolutely no quarantine of the army post against the city. We did not cause unnecessary alarm by publishing daily bulletins, as we thought it more injurious than effective, but the records were open to anybody that wanted to inform himself, and the facts were given to anybody for the asking. We know how the public is inclined to exaggeration and causing panics without reason or cause. Publications of bulletins is without effect in preventing the spread of epidemic cerebro-spinal meningitis, we avoided all such unnecessary agitation, but restricted ourselves to dealing with the cases as they happened, which is actually the only thing we could do, discussed the question scientifically in open meetings of the profession and advised the public in regard to preventive means through individual efforts of the members of the profession.

The fact that San Antonio, the largest city in Texas, had the smallest number of cases, whereas, in all other cities throughout the State, the cases were numbered by the hundreds, speaks best of the manner in which we handled the situation here.

Thanking you for your kind opinion, and asking you to kindly give the above utterance space in your "Red Back" ("Mendacitorium" corner), we remain,

Yours fraternally,

THE BOARD OF HEALTH OF THE CITY OF SAN ANTONIO,

Per DR. S. BURG, Health Officer.

OFFICE OF THE SURGEON,
FORT SAM HOUSTON, TEXAS.

June 16, 1912.

*Dr. S. Burg, Health Officer, San Antonio, Texas.*

DEAR DOCTOR: I am in receipt of yours of June 15th, enclosing a copy of your reply to the scurrilous article in the *New York Medical Record* of May 4th.

I concur in all the statements therein and fully approve of your conduct of the epidemic.

It is to be regretted that a journal of such standing should publish a purported report from "the wife of an army officer" without some further inquiry.

Yours respectfully,

D. M. APPEL,
Colonel, Medical Corps.

---

A felon should be aborted by covering the end of the finger with cotton saturated with alcohol, and then excluding the air by drawing over all a rubber finger cot.—*American Journal of Surgery.*

